# Phase I trial of dose-escalated stereotactic radiosurgery (SRS) boost for unfavorable locally advanced oropharyngeal cancer

**DOI:** 10.1186/s13014-020-01718-w

**Published:** 2020-12-11

**Authors:** Prashant Vempati, Aditya N. Halthore, Sewit Teckie, Zaker Rana, Emile Gogineni, Jeffrey Antone, Honglai Zhang, Mihaela Marrero, Kristin Beadle, Douglas K. Frank, Mohamed Aziz, Doru Paul, Maged Ghaly

**Affiliations:** 1 Department of Radiation Medicine, Northwell Health, Zucker School of Medicine at Hofstra/Northwell, 450 Lakeville Road, Lake Success, NY 11040 USA; 2 Hofstra Northwell School of Medicine, Hempstead, NY USA; 3 Department of Otolaryngology, Northwell Health, Lake Success, NY USA; 4 Department of Hematology/Oncology, Northwell Health, Lake Success, NY USA

**Keywords:** Radiosurgery, SRS, Dose escalation, Oropharynx, Head-and-neck

## Abstract

**Background and purpose:**

Patients with locally advanced oropharynx squamous cell carcinoma have suboptimal outcomes with standard chemoradiation. Here, we evaluated toxicity and oncologic outcomes of dose escalation using radiosurgical boost for patients with unfavorable oropharynx squamous cell carcinoma.

**Materials and methods:**

Between 2010–2017, Thirty four patients with intermediate- or high-risk oropharynx squamous cell carcinoma were enrolled onto this prospective phase I trial. Each patient received concurrent cisplatin and fractionated radiotherapy totaling 60 Gy or 66 Gy followed by radiosurgery boost to areas of residual gross tumor: single fraction of 8 Gy or 10 Gy, or two fractions of 5 Gy each. Primary endpoint was treatment toxicity. Secondary endpoints were local, regional, and distant disease control.

**Results:**

Eleven, sixteen and seven patients received radiosurgery boost with 8 Gy in 1 fraction, 10 Gy in 1 fraction, and 10 Gy in 2 fractions respectively. Acute toxicities include 4 patients with tumor necrosis causing grade 3 dysphagia, of which 3 developed grade 4 pharyngeal hemorrhage requiring surgical intervention. At 24 months after treatment, 7%, 9%, and 15% had grade 2 dysgeusia, xerostomia, and dysphagia, respectively, and two patients remained feeding tube dependent. No grade 5 toxicities occurred secondary to treatment. Local, regional, and distant control at a median follow up of 4.2 years were 85.3%, 85.3% and 88.2%, respectively. Five patients died resulting in overall survival of 85.3%.

**Conclusions:**

This study is the first to report the use of radiosurgery boost dose escalation in patients with unfavorable oropharynx squamous cell carcinoma. Longer follow-up, larger cohorts, and further refinement of boost methodology are needed prior to implementation in routine clinical practice.

*Trial Registration*: Northwell Health Protocol #09-309A (NCT02703493) (https://clinicaltrials.gov/ct2/show/NCT02703493)

## Introduction

Locoregional control rates after curative radiation therapy for patients with oropharynx (OP) squamous cell carcinoma (SCC) unassociated with human papilloma virus (HPV) or those with prolonged tobacco exposure are significantly lower than the 80% local control rates expected for HPV-associated disease [1–3]. Ang et al*.*’s [4] analysis of RTOG 0129 reported a significant difference in 3-year local failure for patients with intermediate-risk OP SCC (14%) when compared to those with high-risk OP SCC (35%). Other studies treating patients with HPV-negative disease with chemoradiotherapy have shown similarly poor outcomes with locoregional failure rates at 2 to 3 years ranging between 33% and 74% [4–7]. Treatment failure in these patients is often confined to the radiotherapy field, suggesting that a subpopulation of tumor cells may be resistant to standard radiation doses, and that a higher therapeutic index may be required for effective local control [7, 8]. Escalating radiation dose by standard techniques, however, can result in significant local morbidity and symptoms such as stroke or cerebrovascular events [9].

We hypothesized that stereotactic radiosurgery (SRS), which affords more precise radiation delivery than intensity modulated radiotherapy (IMRT), may be required to deliver escalated doses to primary disease while avoiding nearby functional structures. Furthermore, preclinical studies suggest that the unique effect of single high dose SRS may be driven by ceramide-mediated apoptosis and alterations of tumor vasculature [8, 10]. To this end, a small number of retrospective studies have reported improved local control in patients with OP SCC receiving SRS boost after IMRT, and their functional and toxicity outcomes were acceptable [11–15].

The basis for this study was to prospectively explore the effect of SRS boost in unfavorable-risk OP SCC, by replacing the standard fractionated cone-down dose to gross disease with a single-fraction or two-fraction SRS dose. The primary endpoint was to determine maximum tolerated dose (MTD) of dose-escalated SRS boost. Secondary objectives included determining dose-limiting toxicity (if the maximum tolerated dose was reached) and measurement of locoregional and distant disease control.

## Materials and methods

Institutional review board (IRB) approval was obtained for this phase I trial in which eligible patients gave written informed consent and were subsequently enrolled. Eligibility criteria included patients with intermediate- or high-risk SCC of the oropharynx, as defined by Ang and colleagues [4]. Patients considered to have intermediate-risk features included: (1) HPV-positive, N2b to N3 tumors and smoking history of at least 10 pack-years; or (2) HPV-negative, T2 or T3 tumors, and smoking history of less than 10 pack-years. Patients at high-risk had: (1) HPV-negative tumors and at least 10 pack-years of smoking history, or (2) T4 disease regardless of HPV status. High-risk HPV subtype positivity was assessed by fluorescence in-situ hybridization analysis, and the expression of EGFR, p16, and p53 was assessed by immunohistochemistry.

### Study treatment

**Fig. 1:**
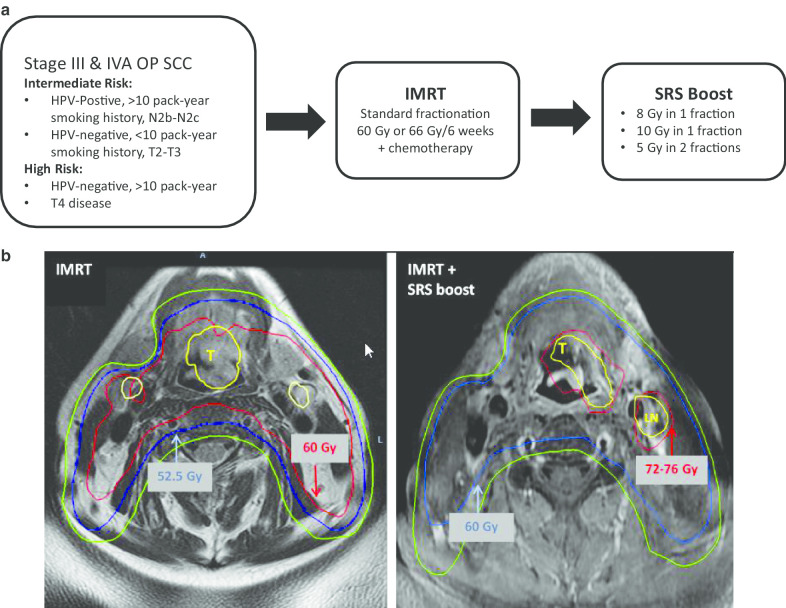
**a** Patient flow through protocol. **b** Example IMRT plan prior to any radiotherapy (left) and SRS plan after completion of IMRT (right) overlaid on a T1 post-contrast MRI sequence

Each patient in the study received fractionated radiation therapy concurrent with intravenous platinum-based chemotherapy either weekly or every three weeks. For safety purposes, and to clearly define SRS-specific toxicities, no systemic therapy was allowed after the final week of fractionated radiotherapy. Radiotherapy was delivered Monday through Friday in 30 daily fractions as follows: areas of gross disease and high risk received 60 Gy or 66 Gy in 2 Gy or 2.2 Gy fractions and areas of low risk received 54 Gy or 52.5 Gy in 1.8 Gy or 1.75 Gy fractions, respectively. An IMRT technique was utilized to spare swallowing organs at risk [1, 16, 17]. One week after IMRT completion, patients received a single fraction SRS boost of 8 Gy corresponding to a total biological effective dose (BED) of 79.1 Gy in 2 Gy equivalent dose fractions assuming an alpha–beta ratio for tumor of 10 Gy (EQD_10/2_). Over time, the boost dose was escalated to a single fraction SRS boost of 10 Gy (corresponding to 83.8 Gy or 76.7 Gy EQD_10/2_) if no dose limiting toxicities developed with the 8 Gy arm according to study design. Lastly, the final seven patients on the protocol received SRS boost 10 Gy in 2 fractions (corresponding to 72.5 Gy EQD_10/2_) after DLTs were identified in the single-fraction arm. Figure 1a diagrams patient flow through this protocol.

### Radiation planning and technique

Patients enrolled in the protocol underwent contrast-enhanced magnetic resonance imaging (MRI) of the neck and non-contrast computed tomography (CT) simulation before IMRT. Figure 1b displays example IMRT and SRS plans for an enrolled patient. For the IMRT portion, the clinical target volume (CTV) receiving 60 Gy or 66 Gy included the primary tumor plus region(s) of grossly involved lymphadenopathy and high-risk subclinical regions with 7 mm expansion. Initial 16 patients (11 of the 8 Gy group and 5 in the 10 Gy in single fraction group) out of the 34 patients were treated to 66 Gy followed by the boost. The rest of the 18 patients were treated with 60 Gy followed by the boost based on the departmental protocol. The CTV receiving 54 Gy or 52.5 Gy included all other regions at risk for harboring microscopic disease. The planning target volume (PTV) was defined as a 3 mm expansion of the CTV. The boost target volume was determined by repeating CT simulation and MRI after the last day of IMRT, and fusing the T1 post-contrast fat suppression MRI sequence to the CT images. Boost gross tumor volume (GTV) included areas of primary and nodal gross tumor delineated on the CT-MRI fusion, and was uniformly expanded by 2 mm to create a boost PTV. In patients whose tumors demonstrated complete response on post-IMRT MRI, the pre-IMRT gross tumor was delineated to form the boost GTV. Two-dimensional kilovoltage imaging was used for set-up and intra-fraction verification prior to delivery of each field. The boost was delivered five working days after completion of IMRT in for all enrolled patients. Patients were evaluated 6 weeks after treatment completion, and followed by imaging every 3 to 4 months for two years and then annually up to 5 years.

Dose constraints for the IMRT portion were applied to the involved pharyngeal constrictor muscles (V60 Gy ≤ 0.5 cc), uninvolved pharyngeal constrictor muscles (D33 < 45 Gy; D15 < 60 Gy; mean dose < 35 Gy), involved supraglottic larynx (SGL) (mean dose < 30 Gy), and uninvolved SGL (mean dose < 18 Gy). Dose constraints for the boost portion included coverage of the boost PTV by the 80% isodose line (ranging between 60% and 90% isodose lines), with “hot spot” kept within the PTV. Organs at risk were specified to receive no more than 30% of the boost dose. The cumulative maximum spine dose for the combined SRS and IMRT treatment was limited to no more than 50 Gy (EQD2). No carotid artery constraints were applied.

### Adverse event reporting

Acute and late adverse events including mucositis, dysphagia, xerostomia, neurologic toxicity, skin toxicities, vascular effects, and constitutional symptoms related to treatment were reported and scored for severity using the NCI Common Terminology Criteria for Adverse Events (CTCAE) version 4.03 [18]. A dose-limiting toxicity (DLT) was defined as any treatment-related CTCAE grade 3, 4, or 5 toxicity that was not present prior to boost. All reported DLTs were verified by the study chair and Data Safety Monitoring Committee before final determination that a DLT did in fact occur. Initial SRS boost dose was 8 Gy with a plan to escalate to the maximum tolerated dose (MTD), defined as the highest dose level at which ≤ 33% of patients experience DLT. The study was designed to be considered complete when either of the following events occurred: (1) the MTD for a cohort was reached, or (2) the highest protocol dose level was treated and tolerated where therapy was likely to be tumorcidal per the determination of investigators.

To assess adverse functional and subjective symptom effects from treatment, patients completed the PSS-HN questionnaire and the M. D. Anderson Symptom Inventory Head and Neck Module (MDASI) core and head-and-neck surveys at baseline, treatment completion, and at routine follow-up appointments over the subsequent 24 months [19, 20].

Percutaneous endoscopic gastrostomy (PEG) tubes were prophylactically placed only in patients who met departmental criteria, which included initial abnormal swallowing pattern as assessed by a speech pathology team and multiple comorbidities. Patients who lost more than 10% body weight during IMRT typically received reactive PEG placement. Overall duration of PEG dependence was calculated from date of boost completion. Radiologic outcomes were assessed by head-and-neck radiologists. Objective response was calculated from assessments performed at baseline, 6–8 weeks after completion of treatment, and during the follow-up period using RECIST version 1.1 criteria.

### Study design

The study was a “7 + 2 + 3 + 3” dose escalation design. For a given SRS boost doselevel, a total of seven to fifteen patients will be enrolled. For a given dose, there will be up to four cohorts of subjects enrolled (Cohorts 1, 2, 3, 4), with maximum sample sizes of 7, 2, 3, and 3, respectively. Patient enrollment in the study, as well as the decision to escalate, add a new cohort, or stop the trial, were restricted by the number of subjects who developed DLTs during their 90-day follow-up. The sample size of this study was not to exceed 45 patients, to allow for a maximum of 3 SRS dose cohorts containing 15 patients each.

### Statistical methods

The primary objective of the study was safety, as measured by development of DLTs during the follow-up period. The secondary objectives, for which we used statistical analysis, was to estimate the failure rates (overall, local, regional, and distant) using standard methods for proportions with their corresponding exact binomial 95% confidence intervals, as well as local, regional, and distant control rates (calculated as 100 minus failure rates). The Fisher’s exact test was used to compare those subjects who received 8 Gy versus 10 Gy for each of the failure rates. Kaplan–Meier curves were used to portray the outcomes. A result was considered statistically significant at the p < 0.05 level of significance. All analyses were performed using SAS version 9.4 (SAS Institute Inc., Cary, NC).

## Results

Between 2010 and 2017, forty-five eligible patients with intermediate- and high-risk OP SCC were screened, and 39 prospectively enrolled. One patient withdrew from the study, two patients died during chemoradiation therapy before the boost phase and two patients were not treated according to protocol (did not receive the boost). Therefore, 34 patients remained eligible for analysis with a median follow-up of 50 months. Patient and treatment characteristics are reported in Table 1. Eleven patients received a single fraction SRS boost of 8 Gy, 16 patients received a single-fraction of 10 Gy and 7 patients received 10 Gy in 2 fractions. Primary OP tumor subsites were tonsil (56%), base of tongue (38%), and pharyngeal wall/soft palate (6%). Ninety-one percent of patients had stage IV cancers, of which T3, T4a and T4b tumors comprised 32%, 15% and 3% of the cohort, respectively. Sixty-seven percent had N2b or N2c tumors. Sixty-two percent had HPV-positive tumors and 94% had over 10 pack-year smoking history (median 35 pack-years). EGFR mutations were overexpressed in 40%, and p53 was underexpressed in 80%. Thirty-four primary and 42 nodal tumors were treated with SRS boost. Mean dose from all treatment courses to ipsilateral parotid, contralateral parotid, supraglottic larynx and superior constrictor muscle were 39 Gy, 25 Gy, 26 Gy and 46 Gy, respectively. Seventy-nine percent of all patients completed three cycles of cisplatin-based chemotherapy with their assigned radiotherapy treatments. Four patients were initially started on cisplatin and switched to carboplatin and paclitaxel during the course of their treatment. One patient’s chemotherapy records from their private medical oncologist were unavailable at the time of this chart review. They were recommended to receive cisplatin.

**Table 1:** Patient and treatment characteristics

Characteristic	No. of patients (%) (N = 34)
Age (mean)	60 years
Smoking history (median)	35 pack-years
Gender	
Male	27 (79%)
Female	7 (21%)
AJCC 7th edition Stage	
III	3 (9%)
IVa	28 (82%)
IVb	3 (9%)
AJCC 8th edition stage	
II (P16 positive)	17 (50%)
III (P16 positive)	4 (12%)
III (p16 negative)	3 (9%)
IVa (p16 negative)	10 (29%)
T Classification	
T1	2 (6%)
T2	15 (44%)
T3	11 (32%)
T4a	5 (15%)
T4b	1 (3%)
N Classification	
N0	1 (3%)
N1	5 (15%)
N2a	5 (15%)
N2b	14 (41%)
N2c	9 (26%)
Primary site	
Tonsil	19 (56%)
Base of tongue	13 (38%)
Pharyngeal wall	1 (3%)
Soft palate	1 (3%)
Mean boost volume	54 cc (range 13–185 cc)
8 Gy SRS boost	11 (32%)
10 Gy SRS boost	16 (47%)
5 Gy × 2 SRS boost	7 (21%)
HPV status	
HPV-positive smokers	21 (62%)
HPV-negative	13 (38%)
Chemotherapy	
Cisplatin	27 (79%)
Cetuximab	1 (3%)
Carboplatin paclitaxel	1 (3%)
Cisplatin followed by carboplatin and paclitaxel	4 (12%)
Unknown	1 (3%)

### Toxicity and adverse events

Grade 1 and 2 post-treatment acute adverse events (within 90 days) included dysgeusia, xerostomia and dysphagia in 88%, 100%, and 85%, respectively (Table 2). Four patients experienced CTCAE grade 3 dysphagia and pain (one patient in the 8 Gy cohort and three in the 10 Gy boost cohort). At 12 months after treatment completion, five patients (3 with tonsillar primary, 2 with base of tongue primary; 4 in the 10 Gy boost with 60 Gy standard fractionation cohort) with HPV-associated tumors had developed grade 3 dysphagia and were found to have radiation-induced oropharyngeal ulceration. Two patients were treated successfully with hyperbaric oxygen. The remaining 3 patients developed grade 4 pharyngeal hemorrhage requiring angiography with embolization followed by mandibulotomy with resection of the pharyngeal wall ulceration and free forearm reconstruction of the defect with microvascular anastomosis. Of the three patients with grade 4 pharyngeal hemorrhage, One patient received two cycles of 50 mg/m^2^/day for 2 days of cisplatin, Second patient started with 80 mg/m^2^ of cisplatin for 2 cycles and was switched to Taxol (50 mg/m^2^) and carboplatin (AUC^2^), and the third patient received taxol (50 mg/m^2^) and Carboplatin (AUC^2^) weekly for 5 cycles. There were no grade 5 toxicities.

**Table 2:** Acute (defined as within 90 days of radiation treatment) and late (greater than 90 days after radiation treatment) toxicities after radiotherapy, as quantified by CTCAE schema

	Acute toxicities	Late toxicities
Dysgeusia		
Grade 0	4 (12%)	4 (12%)
Grade 1	19 (56%)	22 (65%)
Grade 2	11 (32%)	8 (24%)
Grade 3	0 (0%)	0 (0%)
Dysphagia		
Grade 0	1 (3%)	4 (12%)
Grade 1	18 (53%)	11 (32%)
Grade 2	11 (32%)	14 (41%)
Grade 3	4 (12%)	5 (15%)
Pain		
Grade 0	1 (3%)	4 (12%)
Grade 1	8 (24%)	14 (41%)
Grade 2	21 (62%)	14 (41%)
Grade 3	4 (12%)	2 (6%)
Xerostomia		
Grade 0	0 (0%)	0 (0%)
Grade 1	17 (50%)	27 (79%)
Grade 2	17 (50%)	7 (21%)
Grade 3	0 (0%)	0 (0%)

Twenty patients (59%) required PEG placement during their treatments. The rates of PEG dependence at 6, 12, and 24 months after treatment completion were 18%, 9%, and 6%, respectively. Public eating scores were significantly lower in those with feeding tubes compared to those without feeding tubes at all time points after completion of treatment (p = 0.04). Functional outcomes at 24 months included grade 2 dysgeusia (7%), grade 2 xerostomia (9%), and grade 2 dysphagia (15%), and two patients remained PEG dependent.

**Fig. 2:**
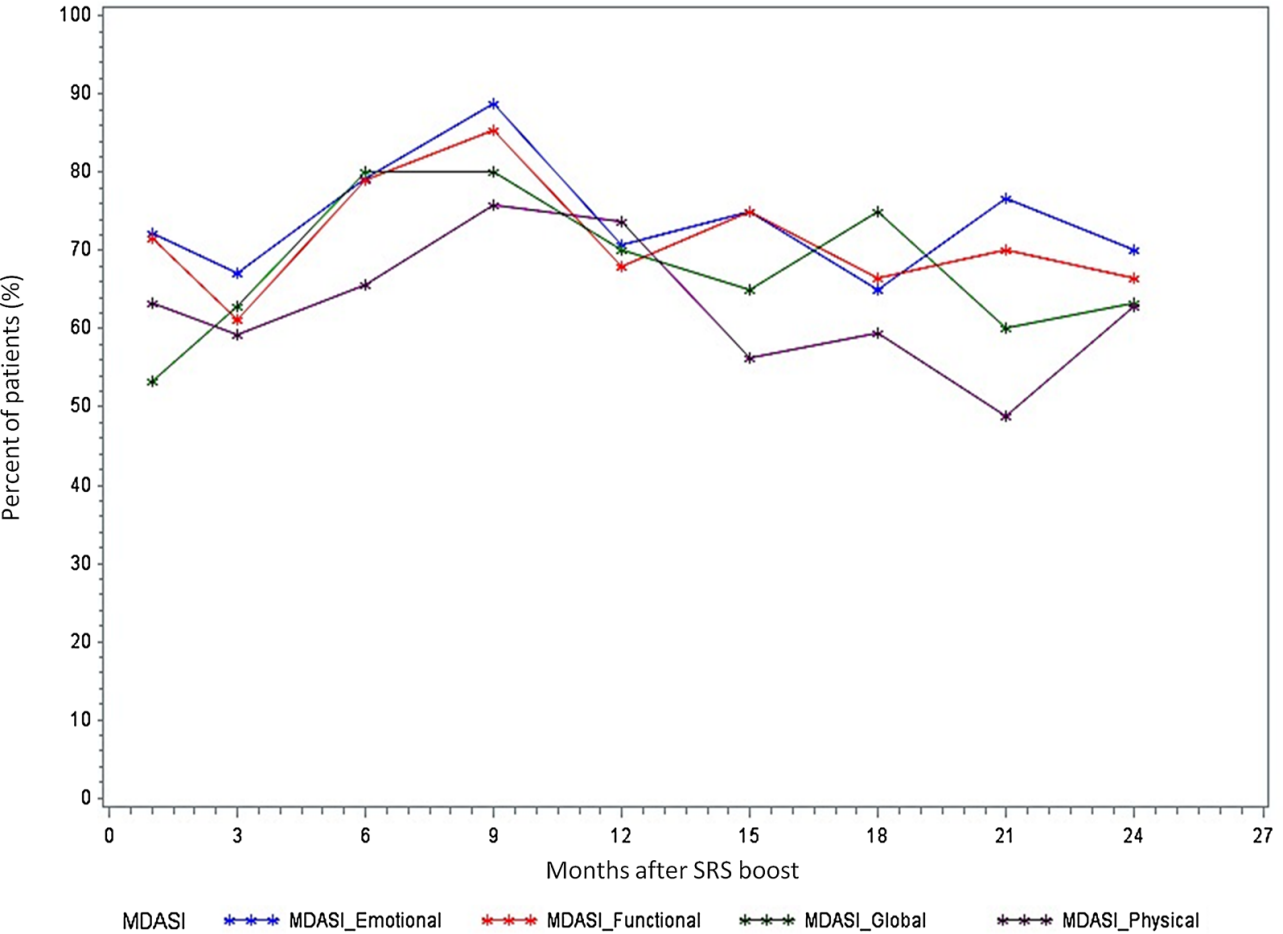
MDASI assessment of 4 quality of life domains over time

Quality of life measures collected from MDASI are displayed in Fig. 2. The most significant impact on quality of life occurred at nine months after radiotherapy completion, with an increase in severity in all four domains around this time, after which a gradual return to baseline was seen. There were no grade 3 or higher persistent long-term toxicities of dysphagia, dysgeusia, and xerostomia were seen in our patient cohort receiving SRS boost. Patients experienced a peak in dysphagia around 9 months, which corresponded to the MDASI scores, but these symptoms decreased in longer-term follow-up. There was a gradual decline in pain score after the 9 month time point to less than pretreatment levels.

While no adaptive planning occurred during IMRT, seventeen patients (65%) had reduction of primary and nodal GTVs after completing 60 Gy. Median primary and nodal GTVs before IMRT were 70 cc and 57 cc, respectively, while median primary and nodal GTVs after IMRT were 59 cc and 51 cc, respectively, as determined from MRI images obtained prior to and on the last day of IMRT. These GTVs were calculated to include gross or residual tumor and peritumoral hyperintense signal on the contrast-enhanced T1, fat suppressed axial MRI sequence. After IMRT, 5 patients (15%) and 4 patients (12%) had complete resolution of their primary tumor and nodal GTV, respectively. In these patients, the pre-IMRT GTVs were used for the boost volume. Mean SRS boost volume was 57.0 cm^3^ (range 16.0–185.0 cm^3^). The median doses delivered to 90% of the target volume (D90) for the 8 Gy and 10 Gy groups were 7.9 Gy and 9.5 Gy, respectively.

**Fig. 3:**
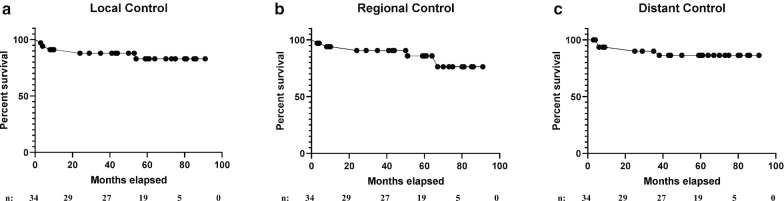
Kaplan–Meier curves of 34 patients for **a** local control after radiation therapy. **b** Regional control after radiation therapy. **c** Distant control after radiation therapy

**Fig. 4:**
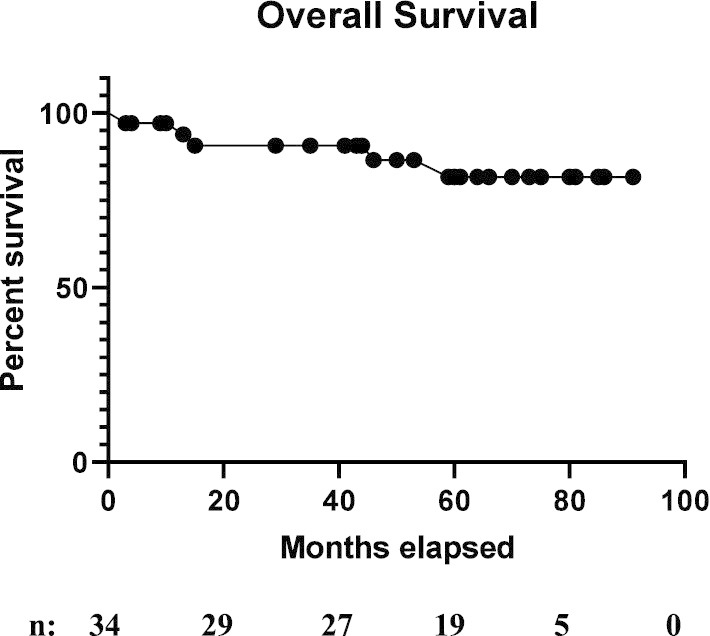
Kaplan–Meier curves of 34 patients for Overall survival after radiation therapy

**Fig. 5:**
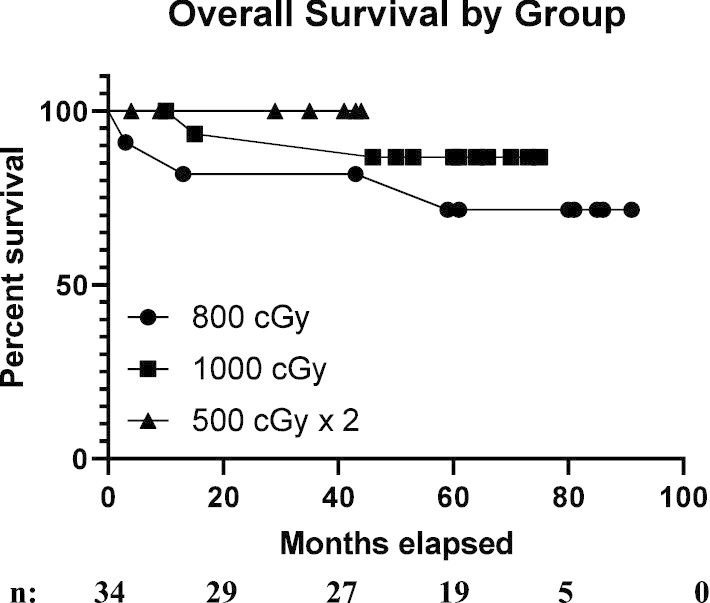
Kaplan–Meier survival curves for the 8 Gy, 10 Gy and 5 Gy in 2 fractions boost cohorts after radiation therapy

After median follow-up of 50 months, local, regional, and distant control were 85.3%, 85.3% and 88.2% (Fig. 3a–c, respectively). There was no significant difference between the 8 Gy and 10 Gy dose groups with respect to local failure (9.1% vs. 0.0%, respectively, p < 0.41), regional failure (18.2% vs. 0.0%, respectively, p < 0.16) and distant failure (0.0% vs. 18.8%, respectively, p < 0.25). Five patients with treatment failures had median smoking histories of 41 pack-years. In the 8 Gy boost cohort, one patient with HPV-associated disease developed locoregional and distant failure and one patient with HPV-unassociated disease had regional failure. In the single fraction 10 Gy boost cohort, three patients had distant failure, all of whom had HPV-associated tumors. Five patients died during the follow-up period, 3 in the 8 Gy in single fraction cohort and 2 in 10 Gy in single fraction cohort. Four patients died within 24 months of treatment conclusion, two patients died due to cardiac events, both in the 8 Gy boost cohort and two patients died in hospice after being admitted for local recurrence. Additionally, one patient died of an unrelated second primary head and neck cancer 59 months after the conclusion of his RT for the initial cancer. Overall survival was 85.3% (Fig. 4). Intention to treat overall survival including all the enrolled patients is 82%. The 2- year survival was 97.1% and 3- year survival was 90.6%. Figure 5 portrays Kaplan–Meier survival curves for the 8 Gy, 10 Gy and 10 Gy in 2 fractions boost cohorts.

## Discussion

Oropharyngeal squamous cell carcinoma are heterogeneous cancers with varied treatment outcomes. There has been a profound movement and success towards treatment de-escalation for HPV positive low-risk OP SCC’s. [21–24] This even led to the change in the latest AJCC 8 staging system. [25] However, there has not been as much enthusiasm for changing the treatment paradigm for intermediate/high risk oropharyngeal cancers. Ang et.al reported in their phase III randomized controlled trial with their follow-up of 4.8 years, 3 year overall survival of 82.4% with HPV positive OP SCC compared to 57.1% for HPV negative disease [4]. This trend persisted with progression free survival, and local regional relapse. However, HPV status is not the only risk factor identified in their analysis of the data. Tobacco smoking status, Tumor T-stage and N-stage were also meaningful and were validated by other retrospective and prospective studies [4]. In Ang et. al’s paper, using a recursive partitioning analysis with smoking status, T and N stages along with HPV status provided us with three risk groupings—Low, intermediate and High risk. 3-year overall survival was 93% low versus 71% intermediate versus 46% high. Intermediate and high risk patients have suboptimal tumor control.

We hypothesized that highly conformal dose escalation using SRS might yield better locoregional outcomes. Standard IMRT techniques protect normal tissues adjacent to tumors but still deliver considerable radiation dose to the uninvolved pharyngeal mucosal sites, sacrificing patient rehabilitation after treatment [1, 2, 26]. SRS is a more precise radiation delivery system that can improve on the therapeutic ratio. Stereotactic radiosurgery (SRS) generally refers to delivery of very focal and conformal dose profiles of radiation with steep dose gradients toward normal tissue, and carrying out the treatment with sub-millimeter accuracy. As the Head & Neck region of the body are routinely immobilized with rigid thermoplastic mask devices which permit for high precision treatment administration, we can use the benefits of SRS. SRS has become a treatment that is well beyond just stereotactic targeting. SRS is about ablative range dose per fraction, accounting properly for errors including motion, careful construction of dosimetry that compacts high dose into the tumor and not normal tissues, and extra careful treatment conduct. This can lead to a dose escalation with a feasible therapeutic ratio.

After a median follow-up of nearly 4.2 years in our study, local, regional and distant controls were excellent. Our overall survival and local control rates were 85%. However, there were increased toxicities in patients who received single fraction SRS boost. The toxicities were mainly seen in the single-fraction boost dose of 10 Gy. As a result of these unacceptable toxicities, study chair and data safety monitoring committee defined single fraction 10 Gy boost dose level as the dose-limiting toxicity (DLT) and the decision was made to alter the boost dose, the last cohort of patients were treated with 10 Gy in 2 fractions With a median follow-up of 29.14 months, there have been no grade 3 or 4 toxicities associated with that cohort. One patient in the 10 Gy in 2 fractions boost cohort was found to have asymptomatic osteoradionecrosis secondary to sialolithiasis in the area of the mandible adjacent to the SBRT boost 2 years after RT. Max point dose to the mandible in the area was 72.42 Gy EQD2. Another patient in the 10 Gy in 2 fractions cohort was found to have a persistent nonhealing biopsy induced ulcer in the region of the initial primary tumor. This was biopsy proven to be not malignant. The patient was treated with Opioid pain medication and hyperbaric oxygen with good relief.

As close to 60% of our patient population was HPV-positive, and our sample size is limited to 34 patients, it was not powered to evaluate the differences between the groups. Ideally, our patient population would be HPV-negative extensive smokers since these tumors are the least responsive to standard of care treatments [27]. Nonetheless, at the time of the study design and initiation, the Ang et al. [25] classification of risk groups for OP SCC was the standard of care, and as such our study used that for risk stratification. All future studies can now be stratified using the AJCC 8th edition staging system.

In our study, we report a relatively low rate of tumor response following IMRT when compared to previously published data in similar cohorts—this is likely due to the fact that response was evaluated immediately after completion of IMRT by MRI rather than the more typical evaluation 10 to 12 weeks after completion of treatment.

While we report comparable local control rates to a previous SRS boost study by Al-Mamgani et al., we also report a higher toxicity profile [11]. In their paper, the authors retrospectively reported on 51 patients with stage I–IV oropharyngeal cancer receiving radiosurgical boost consisting of 3 fractions of 5.5 Gy each after a course of 46 Gy IMRT (67 Gy EQD2). In their cohort of more favorable OP SCC, overall local control was 86% at 2 years, 28% developed mucosal ulceration, and rates of grade 2 or higher dysphagia and xerostomia were 15% and 28%, respectively. In the present study, the highest grade toxicities involved five patients who developed radiation-induced oropharyngeal ulceration at the primary tumor site. Three patients developed grade 4 pharyngeal hemorrhage requiring major surgery. Of note, these 3 patients had locally advanced tonsillar primaries with parapharyngeal extension and increased peritumor vascularity seen on dynamic contrast-enhanced MRI. For each case, the post-IMRT MRI revealed a substantial reduction in tumor volume, but in no case was tumor absent at the time of SRS boost. Tonsillar primaries may therefore require particular caution for radiosurgical boost safety. The single-fraction approach used in our study may explain the severe toxicities observed, which may be mitigated by fractionation of the boost dose. Four of the 5 patients who developed grade ≥ 3 toxicities received a 10 Gy boost in a single fraction (late complication BED 85 Gy EQD3/2). 25% complication rate in the 10 Gy in single fraction was deemed unacceptable, and moving forward, the protocol has been amended to deliver the 10 Gy boost in twice weekly 5 Gy fractions (72 Gy EQD3/2) to avoid late complications by respecting the late BED corresponding to 70 Gy EQD3/2 reported by Fowler et al., while still achieving a higher tumor dose of 72.5 Gy EQD10/2 [28]. There were no grade 3 or 4 toxicities with fractionated SRS boost.

Our study supports further investigation of SRS boost dose escalation in patients with intermediate-high risk oropharyngeal cancers. However, we acknowledge that patient selection must be more stringent and be based on the new AJCC 8th edition [25]. In addition, based on our small study we believe that fractionated SRS may provide the optimal therapeutic index for OP SCC. With further refinement, dose escalation with SRS boost after conventional IMRT and concurrent chemotherapy may present a useful non-invasive alternative to surgical approaches for improving control in OP SCC [29–31].

## Conclusion

This Phase I study assesses and documents toxicity profiles and disease responses in patients with unfavorable, locally-advanced OP SCC receiving primary chemoradiation therapy with a dose-escalated SRS boost. Although single fraction SRS boost after conventional IMRT is promising, Patients with bulkier, more locally invasive tumors or those requiring larger boost volumes demonstrated a greater risk of high-grade toxicity, which may be mitigated in the future with a fractionated stereotactic approach. While studies with longer follow-up and larger cohorts are needed, fractionated radiosurgical boost after conventional concurrent chemoradiation may provide an alternative to conventionally fractionated concurrent chemoradiation alone in patients with unfavorable OP SCC.

## Data Availability

The datasets during and/or analysed during the current study available from the corresponding author on reasonable request.
